# The role of neuropsychological mechanisms in implementation intentions to reduce alcohol consumption among heavy drinkers: a randomized trial

**DOI:** 10.1007/s10865-019-00078-5

**Published:** 2019-08-01

**Authors:** Elly McGrath, Rebecca Elliott, Tim Millar, Christopher J. Armitage

**Affiliations:** 1 Faculty of Biology, Medicine and Health, University of Manchester, Manchester, UK; 2 Division of Psychology and Mental Health, Manchester Centre for Health Psychology, Manchester Academic Health Science Centre, United Kingdom and NIHR Manchester Biomedical Research Centre and NIHR Greater Manchester Patient Safety Translational Research Centre, University of Manchester, Manchester, UK; 3 Neuroscience and Psychiatry Unit, G.708 Stopford Building, University of Manchester, Oxford Road, Manchester, M13 9PT UK

**Keywords:** Implementation intentions, Alcohol, Heavy drinking, Behaviour change

## Abstract

Implementation intention formation, which involves identifying triggers and linking them with coping strategies, has proven effective at reducing alcohol consumption in general populations. For the first time, the present study tested the ability of implementation intentions to reduce alcohol consumption among heavy drinkers and to explore potential neuropsychological mechanisms. At baseline, participants were randomized to form implementation intentions or to an active control group. There was a 5.7 unit (1 unit = 10 ml or 8 g ethanol) per week reduction ([95%CI 0.15, 11.19], *p* = 0.048) in alcohol consumption at 1 month follow-up among participants who formed implementation intentions, which was significantly more than controls F(1, 91) = 3.95, *p* = 0.048, a medium effect size (d = 0.47, Cohen, 1992). No significant differences in performance on the neuropsychological tasks were found between groups. The present study demonstrates for the first time that implementation intentions reduce alcohol consumption among heavy drinkers.

**Electronic supplementary material:**

The online version of this article (10.1007/s10865-019-00078-5) contains supplementary material, which is available to authorized users.

## Introduction

Currently, it is estimated that 31% of men and 16% of women drink in excess of UK Government guidelines (Office for National Statistics, 2016) and research suggests that excessive drinkers want to decrease their drinking but show a lack of engagement with formal interventions that are often face to face and can last several weeks (Aalto et al., 2001; Murgraff et al., 2006). Implementation intention formation offers one possible solution that can be delivered remotely and takes fewer than 5 min to complete. Implementation intention formation involves identifying triggers and linking them with coping strategies (Gollwitzer, 1999). They can be self-generated on a single occasion without the help of a health care professional and have been shown to exert medium-to-large effects on reducing alcohol consumption within the general population (Armitage, 2009; Hagger et al., 2012). In contrast, current brief alcohol interventions delivered in clinical populations consist of up to five sessions face-to-face and yield only small effect sizes (O’Donnell et al., 2014, d = 0.15; Platt et al., 2016, d = 0.23). Implementation intention formation therefore has great potential for reducing alcohol consumption among heavy drinkers at low cost and with high public health “reach”. The aim of the present research is to test for the first time whether implementation intentions could reduce alcohol consumption among heavy drinkers and to understand what the mechanism of action might be.

Implementation intention formation involves creating an “if–then” plan whereby the user specifies a rule, such as “If I find myself in such a situation, then I will/will not do…”. Meta-analyses show that these plans aid critical cue detection and reduce impulsive unhealthy processes by providing well-planned alternatives (Webb & Sheeran, 2008) as well as exert strong effects on behaviour, reported as d = 0.65 overall (Gollwitzer & Sheeran, 2006). In terms of reducing alcohol consumption, similar effect sizes from implementation intention-based interventions have been found in general populations (people who drink alcohol irrespective of whether or not they were drinking to excess) (Armitage, 2009, d = 0.67; Hagger et al., 2012, d = 0.55; Murgraff et al., 2006, d = 0.68), but not yet among heavy drinkers.

Implementation intentions are hypothesised to bring about behaviour change through changes in cognition, but currently there is no research that has examined the ability of implementation intentions to compensate for the cognitive deficits associated with excessive alcohol use (Field & Cox, 2008). Dual process theories suggest that addictive behaviours are maintained through lapses in executive control, which lead to greater attention to alcohol stimuli and more impulsive behavioural responses. There is an abundance of evidence for the role of increased levels of impulsivity in heavy drinkers (Field et al., 2007; MacKillop et al., 2011), as well as decreased executive control (van Hemel-Ruiter et al., 2015) and biased attention for alcohol-related stimuli (Field & Cox, 2008). It is likely that these mechanisms also interact with and influence each other in a relationship that helps to maintain heavy drinking (Carbia et al., 2018).

Implementation intentions have been shown to improve bottom-up control over action (Gollwitzer & Sheeran, 2006) and so implementation intentions should: (a) improve deficits in executive functioning that are associated with weaker links between intention to change and poorer planning skills (Mullan et al., 2011) by providing specific and tailored plans; (b) reduce impulsivity by promoting healthier choices, which allows inhibition of unhelpful responses (Gollwitzer & Sheeran, 2006; Parks-Stamm et al., 2007); and (c) highlight attentional bias by identifying a critical cue or situation that is commonly linked to the unhealthy behaviour the user would like to change (Achtziger et al., 2012). However, evidence in support of the full causal pathway has proven elusive, with many studies testing whether implementation intentions improve cognitive performance or behaviour, but rarely testing whether improvements in cognitive performance caused by implementation intentions subsequently change behaviour (Gollwitzer & Sheeran, 2006; but see Armitage, 2016).

One novel way to investigate these processes is to utilise existing neuropsychological tasks targeting these areas of cognition in combination with implementation intentions to investigate their effect on alcohol consumption. Neuropsychological tasks are useful due to their validity and ease of replicability. They are used extensively in addiction research and allow for comparisons across tasks that can reveal the nature of dual process relationships between cognitive deficits that maintain behaviours (Barkby et al., 2012; Fernandez-Serrano et al., 2010; Jones et al., 2013). Implementation intention studies that have previously attempted to investigate mechanisms of change have assessed convenience samples of students and have involved novel computer-based tasks with unknown validity and reliability (Gollwitzer, 1999; Webb & Sheeran, 2007). Further, these mechanistic studies have not investigated whether the effects on the mechanism carry forward to subsequent behaviour (Wieber et al., 2015). The use of neuropsychological tests that are validated, reliable and are linked to brain mechanisms should provide more definitive answers. The aim of the present study is therefore two fold; to apply implementation intentions to a heavy drinking population and to investigate which cognitive mechanisms may underpin their effects. This study will use a series of neuropsychological tasks that have been shown to reliably measure facets of executive functioning, impulsivity and attentional bias to investigate implementation intentions and their effect on alcohol consumption in a population of heavy drinkers.

## Study hypotheses

*H1* Heavy drinkers randomised to form implementation intentions will show significant reductions in alcohol consumption at 1 month follow-up compared to those undertaking a control task.

*H2* Heavy drinkers randomised to form implementation intentions will show significantly improved performance on all neuropsychological tasks compared to those undertaking a control task at immediate and 1 month follow-up.

## Method

This protocol was approved by the South East Coast—Brighton and Sussex Research Ethics Committee (REC Ref: 15/LO/0835). This trial was registered (ISRCTN:14874035) and is available at https://www.isrctn.com/ISRCTN14874035. Sessions were conducted at premises belonging to the University of Manchester or Arrowe Park Hospital, Wirral.

### Participants

92 participants aged 18–65 (mean age = 25.6, SD = 8.72, 58.7% female) were recruited from across the North West of England via online advertisements and posters at testing premises (Table 1). The sample size for this study was determined using a power calculation based on a meta-analysis of implementation intentions that calculated their overall effect on goal achievement (d = 0.65), to have a power of 0.95, alpha value of 0.05 and to include a drop-out rate of 10%. Recruitment occurred from October 2015 until November 2017. Participants were recruited as “heavy drinkers” if they self-reported consuming 30 + (male) or 21 + (female) units of alcohol per week. In the UK 1 unit represents 10 ml or 8 g of ethanol. These limits were based on UK Government advice on alcohol consumption (at the time of study commencement—October 2014) that women should consume no more than 2–3 units and men 3–4 units of alcohol per day with two alcohol-free days a week (House of Commons Science and Technology Committee, 2004). Thus, our sample was consuming at least 50% more units of alcohol per week than recommended by the UK government. Participants were also required to indicate that they were interested in reducing their alcohol consumption.

**Table 1:** Baseline demographic information

	Experimental mean (SD), *n *= 50	Control mean (SD), *n *= 42	*t*	*df*	*p*
Age	26.42 (9.66)	24.64 (7.47)	− .973	90	0.333
BMI	23.13 (3.14)	23.70 (3.41)	.825	90	0.411
Alcohol units per week	35.32 (13.62)	32.76 (9.06)	− .874	90	0.385

	Experimental count	Control count	*X*^2^	*df*	*p*
Male	23	15	.996	1	0.318
Female	27	27			

**Fig. 1:**
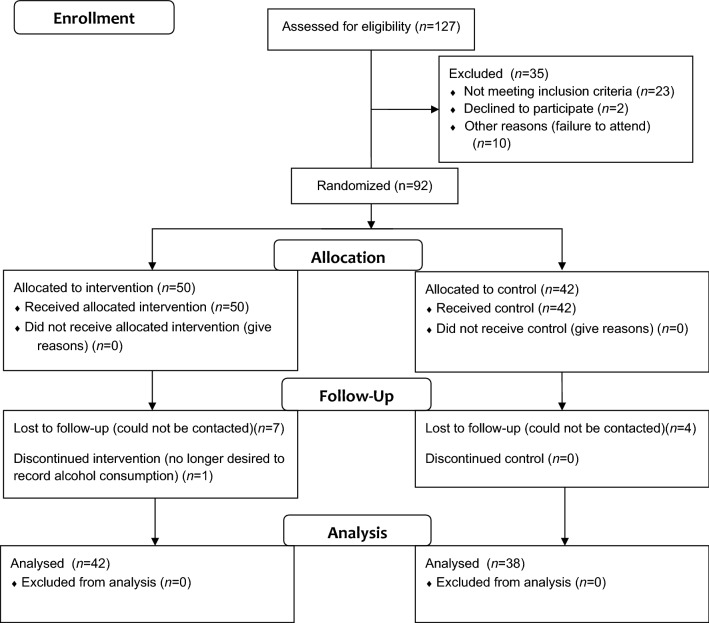
Flowchart of study participants

Following informed consent, an alcohol breath test and drug urine screen was obtained. The urine screen tested for amphetamines, barbiturates, cocaine, opiates, cannabinoids and benzodiazepines. A positive result meant that the participant would be excluded from completing the study on that day only and not the study as a whole. Positive results for cannabinoids were allowed given the long half-life of cannabinoid metabolites. No participants were excluded due to positive drug or alcohol screens. One baseline appointment was rescheduled due to a positive alcohol breath screen. Full CONSORT participant flow is detailed in Fig. 1. Demographic information was collected and an assessment of drug and alcohol history, including the SCID (Structured Clinical Interview for DSM-IV) for dependence history, was conducted. Exclusion criteria from this interview included: use of psychoactive prescription medications, such as those with anti-depressant or anxiolytic properties; a history or presence of a neurological diagnosis; clinically significant head injury; neuroendocrine disorder, including impaired thyroid function and steroid use; current or past substance dependence; current or past psychosis, bipolar disorder or eating disorder; and any current axis I or II disorder, including depression and anxiety. One participant was excluded due to past alcohol dependence and one participant was excluded due to a past eating disorder.

Participants then completed a 2 h testing session comprising a timeline follow-back assessment of alcohol consumption, personality measures and neuropsychological testing. All neuropsychological tests were computerised and were presented to participants on a laptop with a 1600 × 900 pixel screen and a separate mouse. Questionnaires and the implementation intention formation or control task were delivered in sealed opaque envelopes. Prior to study commencement a researcher who was independent of the study team used a random number table to sort opaque envelopes that contained the intervention and control materials into a random order. The researcher who recruited participants was thus blind to intervention allocation. Participants performed the tests in a designated quiet testing room, with comfort breaks as required.

### Baseline

*Timeline Follow*-*back* (Sobell & Sobell, 1992). The researcher used a Timeline Follow-back assessment to record alcohol consumption for the 28 days prior to the appointment. Participants retrospectively recalled their alcohol consumption over the 28 days by filling in calendar with the researcher, using key dates and events as memory aids to help recall. Where units were unknown to the participants as much detail as possible about the alcohol consumed (including type of alcohol, volume and brand) was recorded and later converted to units per day. Participants also confirmed whether this was typical of their usual drinking behaviour. This method has test–retest reliability of r = 0.85.

### Neuropsychological testing

All of the following tasks were programmed using PsychoPy version 1.84.2.

1. Sternberg Task (Assessing executive function)

**Fig. 2:**
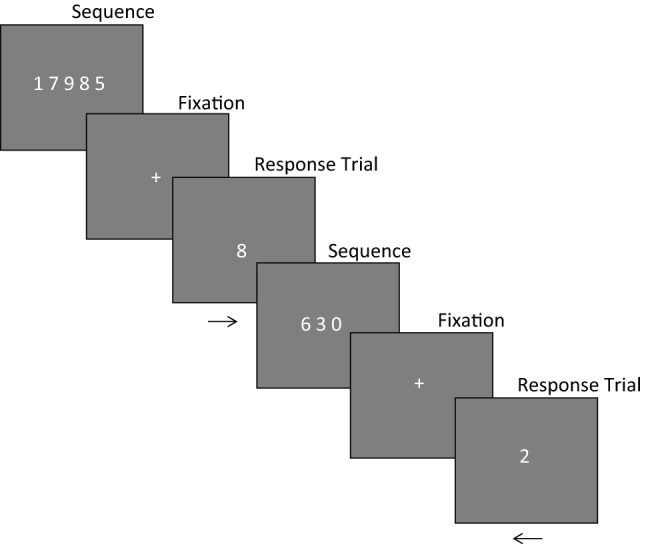
Sternberg task example trials

In this task participants were instructed to memorise a sequence of numbers that would appear on a screen. Trials started with a sequential presentation of anywhere from one to six numbers. Participants would then be presented with a single number and had to indicate whether that number was in the previous sequence by pressing the right cursor key, or was not in the previous sequence by pressing the left cursor key (Fig. 2).

2. Alcohol Stroop Task (Assessing attentional bias)

Participants were told that the first task would involve identifying the colours of a set of words. Firstly, participants were asked to identify the colour of different words by pressing four different keys (“A” for red, “S” for blue, “K” for yellow and “L” for green). The modified Stroop was then administered with alcohol (20 words), emotive (20 words) and neutral (20 words) words presented in a counterbalanced order. Each word was presented in every colour once, meaning that participants were presented with 240 words in total. Responses and response times were recoded.

3. Delay Discounting Task (Assessing impulsivity)

Participants were presented with five sets of 20 either/or monetary choices. The amount of money was adjusted across successive questions (trials) presented to the participants on the computer screen until an indifference point was calculated. Within each session, indifference points for five different time points were calculated: from now to 1 day, 1 week, 1 month, 1 year. Monetary amounts that participants were asked to choose between could be between £100 and £0 in £5 gradients. Programming mimicked that used in Richards et al.’s version of the delay discounting task (Richards et al., 1999).

4. Standard Stop Signal Task (Assessing Impulsivity)

Participants were instructed to categorise circles into one of two groups by pressing the “C” or “M” key. They were told that on some trials they would hear a “beep” when the circle appeared, and that on those trials they should inhibit their response. Participants were further asked to complete the task as fast as possible, without making mistakes.

5. Alcohol Stop Signal Task (Assessing impulsivity)

In this version of the task rather than categorise arbitrary stimuli, participants were required to categorise whether an image was alcohol-related or water-related. They received the same instructions as the standard stop signal task above.

6. Alcohol Approach-Avoidance Task (Assessing attentional bias)

**Fig. 3:**
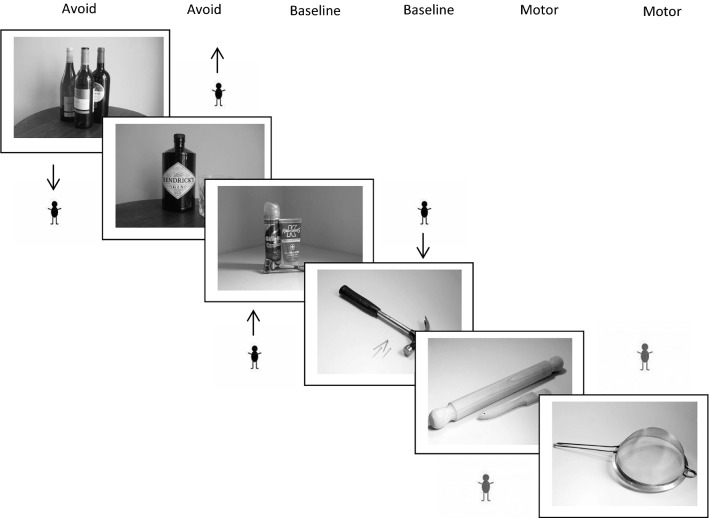
Approach avoidance task example trials

This task was adapted from a task used by Field and colleagues (Barkby et al., 2012; Field et al., 2008, 2011) and a similar fMRl study of automatic approach to cannabis cues by Cousijn et al. (2012). In this task, participants were instructed to categorise images as being either alcohol-related or neutral using an “Approach” or “Avoid” response. This was represented by the movement of a small manikin figure (match stickman) either toward (Approach) or away from (Avoid) images, which participants controlled by pressing the up or down arrow keys (Fig. 3).

### Questionnaires

1. The Self-Regulation Questionnaire (Armitage, 2009; Sniehotta et al., 2006)

Participants completed an adaptation of Sniehotta et al.’s self-regulatory questions. These questions have been shown to have a good internal reliability averaging at α = 0.85. Total scores were calculated to assess self-regulation of alcohol behaviour.

2. The Approach Avoidance of Alcohol Questionnaire (AAAQ) (McEvoy et al., 2004)

Participants completed the “right now” version of this questionnaire scoring 14 statements about their attitudes towards alcohol. Scores on the three subscales were calculated: Inclined/indulgent assessing mild approach inclinations, Obsessed/compelled assessing intense approach inclinations and Resolved/regulated assessing avoidance inclinations. These subscales have high levels of internal consistency (α = 0.90, 0.86, and 0.72).

3. The Self-Report Behavioural Automaticity Index (SRBAI) (Gardner et al., 2012)

Participants scored 4 statements on the alcohol consumption SRBAI, which described their habits towards alcohol in general. These items have been shown to have a minimum internal reliability of α = 0.92. Total scores were calculated to reveal automaticity towards alcohol.

### Intervention and control conditions

Volitional help sheets have been used previously to help participants form implementation intentions targeting a variety of behaviours, including alcohol consumption (Armitage, 2008, 2009; Armitage & Arden, 2010, 2012; Armitage et al., 2016). Participants in both the intervention and control conditions received identical paper volitional help sheets during the intervention/control element of the study. Volitional help sheets consist of a single A4 sheet of paper consisting of two columns of 10 critical situations and 10 appropriate responses, but with different instructions depending on allocation to control versus intervention condition. The volitional help sheets were delivered in packs together with the questionnaire and numbered prior to randomisation and the researcher was blind as to which numbers related to which condition. Both intervention and control packs contained a sheet with some examples of alcohol units and a list of “situations” and “solutions” (see supplementary materials). The intervention group were instructed to link specific situations with specific solutions and thus form implementation intentions. The control group were instructed to tick all situations and solutions that applied to themselves, but did not link situations and solutions together thereby undertaking a control task equivalently active to the intervention group, but missing the active ingredient. This method has been used successfully before to reduce alcohol consumption (Armitage, 2009; Armitage & Arden, 2012).

### Post-intervention

Neuropsychological tasks were repeated by all participants as an immediate follow-up.

### Online questionnaire battery

Participants were then given a web link at the initial appointment and were asked to complete the UPPS impulsive behaviour scale (Whiteside & Lynam, 2001), Beck Depression Inventory (Beck et al., 1996), State Trait Anxiety Inventory (Spielberger, 2010) and CAGE questionnaire (Mayfield et al., 1974).

### One month follow-up

All participants were followed up for a period of 1 month by telephone interview or email, depending on their own preference, to complete a further 28 days timeline follow-back so as to ascertain any changes in their drinking behaviour. Participants who were recorded as drinking fewer units than 150% of the government recommended guidelines (meaning that they would no longer be classified as heavier drinkers) were recorded to establish the potential clinical significance of the findings. All participants completed all questionnaires and neuropsychological tasks from the baseline appointment once more.

### Data analysis

Data were analysed using SPSS. T-tests and Pearson’s Chi square tests were used to assess potential baseline group differences on demographic information. Compliance checks were conducted on all data prior to data entry. An analysis of covariance (ANCOVA) was used to assess the alcohol consumption of all heavy drinking participants at one-month follow-up, controlling for prior drinking. Univariate analyses of variance (ANOVA) were used to assess potential group differences on the baseline questionnaire measures. Mixed design, repeated measures analyses of variances were used to assess performance on the neuropsychological tasks, in order to reveal any changes in performance from baseline to post-intervention and 1 month follow-up. For the Approach Avoidance Task, approach bias scores were calculated by subtracting mean avoid trial reactions time from mean approach trial reaction times. For the Delay Discounting Task, indifference points were calculated at each delay (1, 7, 28 and 365 days). These were taken as the last “smallest sooner” option chosen, meaning the last smallest immediate reward before participants switched to the larger delayed reward of £100. To analyse the delay discounting data, we followed the approach laid out by Reed et al. (2012). This approach uses area under the curve (AUC) methodology (Myerson et al., 2001) to generate values, with higher AUC values indicative of less steep discounting of the delayed reward and therefore reduced impulsivity. AUC values were calculated separately for baseline and follow-ups. Any effects of significance were further investigated using post hoc tests and independent samples t-tests where appropriate. Where participants were lost between baseline and follow-up this study employed a last observation carried forward technique where it was assumed that participants who dropped of the study did not change.

## Results

### Sample characteristics

Analyses revealed that groups did not differ significantly at baseline on alcohol consumption, age, sex or BMI. Mean alcohol consumption at baseline was 34.16 units per week (SD = 11.77) and ranged from 30.00 to 67.42 (male) and 22.50–94.5 (female). Full details are included in Table 1. Multivariate analyses of variance were conducted to check for any group differences on the questionnaire measures conducted at baseline. No significant differences were found (*p* values > 0.67). 80 participants completed the 1 month-follow up. This study used intention to treat (ITT) analysis, which means that all participants who were enrolled and randomly allocated to a treatment are included in the analysis and are analysed in the groups to which they were randomized.

### Alcohol consumption results

Group differences in weekly alcohol consumption at one-month follow-up, measured in units from the Timeline Follow-back (TLFB), were assessed using a univariate analysis of covariance (ANCOVA). This used a between subjects factor of group and weekly alcohol consumption for the month prior to baseline appointment as the covariate. The main effect of group was statistically significant (F(1, 91) = 3.95, *p* = 0.048). This was a medium effect size of d = 0.47. The mean difference between groups at the one-month follow-up was 5.65 units per week [95%CI 0.15, 11.19], *p* = 0.048. Full details are included in Table 2.

**Table 2:** Effects of implementation intentions on alcohol consumption in units per week between pre-intervention and 1 month follow-up

	Implementation intentions, *n *= 50		Control, *n *= 42	
	*M*	*SD*	*M*	*SD*
Pre-intervention	35.32	13.62	32.76	9.06
One month follow-up	29.95	14.17	32.72	14.54

Mean values are raw scores and unadjusted for pre-intervention alcohol consumption. There was a significant condition × time interaction, F(1, 91) = 3.95, *p* = 0.048, d = 0.47. Mean difference between groups at the 1 month follow-up was 5.65 units per week [95%CI 0.15, 11.19], *p* = 0.048

Of those participants who completed the one-month follow up, 23.6% of the control group were drinking fewer units than 150% of the government recommended guidelines (meaning that they would no longer be classified as heavier drinkers) whereas significantly more (52.3%) of the intervention group were drinking less than 150% of the government recommended guidelines (*Χ*^2^ (2, *n *= 77) = 5.90, *p* = 0.021), thus providing evidence for clinical significance (Table 3).

**Table 3:** Counts of participants drinking on average lower than 150% of the government recommended weekly guidelines at 1 month follow-up

	Control count (total)	Experimental count (total)	*Χ*^2^	*df*	*p*
Male	3 (13)	10 (20)			
Female	6 (25)	12 (22)			
Total	9 (38)	22 (42)	5.895	1	0.021

### Neuropsychological task results

For the Sternberg (measuring reaction times and errors), Standard Stop-Signal Task (reaction times and errors), Delay Discounting Task (AUC values) and Approach Avoidance Task (Approach Bias scores) separate 3 × 2 mixed design analyses of variance (ANOVA) were conducted with a within subjects factors of time (baseline, post-intervention, 1 month follow-up) and a between subjects factor of condition (implementation intention or control). There were no significant interactions (*p* values > 0.10).

For the Alcohol Stop Signal Task a 3 × 2 × 2 mixed design analyses of variance (ANOVA) was conducted with within subjects factors of time (baseline, post-intervention, 1 month follow-up) and trial type (alcohol or water) and a between subjects factor of condition (implementation intention or control) on mean RTs. All interactions were non-significant (*p* values > 0.15).

For the Stroop Task a 3 × 3 × 2 mixed design analyses of variance (ANOVA) was conducted with within subjects factors of time (baseline, post-intervention, 1 month follow-up) and list type (alcohol, emotive or neutral) and a between subjects factor of condition (implementation intention or control) on mean RTs. No significant interactions were found (*p* values > 0.17).

## Discussion

The principal finding from the present study was that implementation intention formation was effective in significantly reducing alcohol consumption by on average 5.7 units per week among heavy drinkers. The magnitude of our effect on reducing alcohol consumption among heavy drinkers is comparable to that observed in some general population samples (people who drink alcohol irrespective of whether or not they were drinking to excess) (Armitage, 2009; Hagger et al., 2012). Importantly, these effects are also larger than those found in current brief alcohol interventions used in clinical services (O’Donnell et al., 2014). This implies that the volitional help sheet could be used with low cost and high public health “reach” to reduce the alcohol consumption of heavy drinkers and could even be more effective than current options available in the UK healthcare system for heavy drinking patients. Presumably a volitional helpsheet for use in treatment would be delivered face-to-face and so may even see more pronounced effects than those reported in the present study. Further testing is needed to confirm this, and it would also be valuable in future research to see whether formats beyond paper-and-pencil could be used to further improve the potential reach of the volitional help sheet.

Contrary to predictions, but consistent with previous studies, we were unable to identify the mechanism of effect despite our novel use of neuropsychological testing. Previous mechanistic studies of implementation intentions have failed to investigate whether the effects on the mechanism carry forward to subsequent behaviour (Webb & Sheeran, 2007; Wieber et al., 2015) and so there is a question-mark over the current hypotheses regarding how implementation intentions operate. Heavy drinkers have previously shown consistent evidence of cognitive deficit on the neuropsychological tasks used in this study (Field et al., 2008; Looby et al., 2018; Wiers et al., 2015), and so it is unlikely that the results are due to the participants having no deficits to begin with. However, it could be that implementation intentions work through areas of cognition that were not measured by the questionnaires or neuropsychological tasks used in this study. It would therefore follow that the neuropsychological tasks themselves would not detect the specific changes implementation intentions may be eliciting. Neuropsychological tasks such as the stop signal task measure impulsivity at a general level and impulsivity as a concept is wide ranging and can operate at a conscious and unconscious level. Conversely, implementation intentions are a specific and targeted intervention. Future research should therefore focus on ways to assess changes in cognition with greater sensitivity. One suggestion for this would be a similar design to present study, but instead using an alcohol-relevant IAT with lexical stimuli drawn from the volitional helpsheet.

This is especially important when considering that implementation intentions have been effective at enacting behaviour change that can last months, and even years (Conner et al., 2018; Conner & Higgins, 2010). Recently it has also been proven that implementation intentions can overcome the effects of habits outside of the laboratory (Epton & Armitage, 2017), which would be particularly useful in preventing more serious addictive disorders from developing. Of course, further research is needed into whether implementation intentions would be viable for clinically dependant populations, when considering that implementation intentions are most effective when the individual is highly motivated to change their behaviour (Hagger & Luszczynska, 2014; Webb et al., 2009). Future research should also consider that clinically dependant patients often present with distinctly different patterns of cognition (Barkby et al., 2012; Czapla et al., 2016) and so would likely require the content of the volitional helpsheet to be tailored, which has been successfully done for other patterns of drinking (Arden & Armitage, 2012; Armitage, 2008, 2015; Conner et al., 2018). If these considerations were taken into account, there is the possibility that similar effects could be found in a clinically dependant population.

### Limitations

There are some limitations to this study design that are of note. Firstly, in order to maintain blindness, the volitional helpsheet was completed by participants out of view of, and without guidance from, the researcher. If the results of this research are to be used in order to develop future alternative treatment options for heavy drinkers it would be useful to investigate whether guidance from a healthcare professional would impact the effects of the volitional helpsheet. Secondly, alcohol consumption was only followed up for a period of 1 month. Although implementation intentions have previously shown effects on other addictive behaviours that have lasted much longer (Conner et al., 2018; Conner & Higgins, 2010), future research should establish whether the effects found in this study would be sustained for similar lengths of time in heavy drinkers.

## Conclusions

In conclusion, the present results provide strong evidence for the use of implementation intentions for reducing alcohol consumption in heavy drinkers. Despite a lack of evidence for general neuropsychological mechanisms underlying these effects, the clinical importance of these effects and the impact they could have on the health of heavy drinkers is not to be underestimated. Future research should focus on utilising these in a population that requires brief and specific interventions.

## Electronic supplementary material

Below is the link to the electronic supplementary material.

Supplementary material 1 (DOCX 151 kb)
