# Supplementary material for: The role of neuropsychological mechanisms in implementation intentions to reduce alcohol consumption among heavy drinkers: a randomized trial
Source: J Behav Med. 2019 Aug 1;43(4):576–86. doi: 10.1007/s10865-019-00078-5 (PMC7366587; doi:10.1007/s10865-019-00078-5)
Supplement: Supplementary file 1 — Supplementary material 1 (DOCX 151 kb) [file 10865_2019_78_MOESM1_ESM.docx]

*Supplementary Materials****:*** *Volitional Help Sheet for Moderate Alcohol Consumption*

We want you to plan to drink within the government’s recommended levels. This is 2-3 units a day for women and 3-4 units a day for men. Please see the unit guide below. Research shows that if people can spot situations in which they will be tempted to drink more than is recommended and then link them with a way to overcome those situations, they are much more likely to be successful in avoiding excessive drinking.

*Experimental Instructions:* On the left hand side of the page below is a list of common situations in which people feel tempted to drink; on the right hand side of the page is a list of possible solutions. For each situation that applies to you personally (left hand side), please draw a line linking it to a solution (right hand side) that you think might work for you. Please draw a line linking one situation to one solution at a time, but make as many (or as few) situation-solution links as you like.

*Control Instructions:* On the left hand side of the page below is a list of common situations in which people feel tempted to drink; please tick all those that apply to you personally. On the right hand side of the page are a series of possible solutions; please tick all those that apply to you personally. Tick as many or as few situations and solutions as you like.


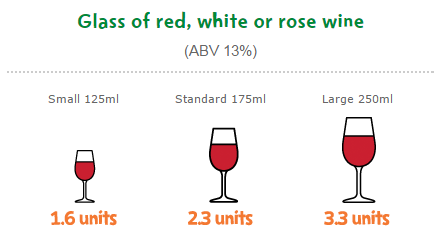

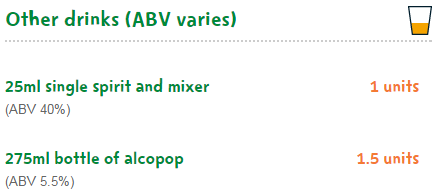

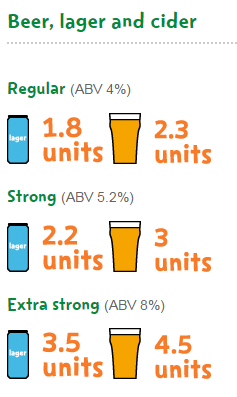


| **Situations** | **Solutions** |
| --- | --- |
| ⬜ If I am tempted to drink more than is recommended when I am excited | ⬜ then I will avoid situations that encourage me to drink |
| ⬜ If I am tempted to drink more than is recommended when I am with others who are drinking a lot | ⬜ then I will do something nice for myself for making efforts to change |
| ⬜ If I am tempted to drink more than is recommended when things are not going my way and I am frustrated | ⬜ then I will do something else instead of drinking |
| ⬜ If I am tempted to drink more than is recommended when I am really happy | ⬜ then I will seek out someone who listens when I want to talk about my drinking |
| ⬜ If I am tempted to drink more than is recommended when my friends push me to keep up with their drinking | ⬜ then I will seek out social situations where people respect the rights of others to not drink |
| ⬜ If I am tempted to drink more than is recommended when I am feeling depressed | ⬜ then I will stop to think about how my drinking is hurting people around me |
| ⬜ If I am tempted to drink more than is recommended when I am having fun with friends | ⬜ then I will remember the information that people have personally given me on the benefits of quitting drinking |
| ⬜ If I am tempted to drink more than is recommended when other people encourage me to have a drink | ⬜ then I will remember that warnings about the health hazards of drinking have an emotional effect on me |
| ⬜ If I am tempted to drink more than is recommended when I am offered a drink by someone | ⬜ then I will tell myself that if I try hard enough I can keep from drinking |
| ⬜ If I am tempted to drink more than is recommended when I am feeling shy | ⬜ then I will think about the type of person I will be if I am in control of my drinking |
